# A bias evaluation checklist for predictive models and its pilot application for 30-day hospital readmission models

**DOI:** 10.1093/jamia/ocac065

**Published:** 2022-05-17

**Authors:** H Echo Wang, Matthew Landers, Roy Adams, Adarsh Subbaswamy, Hadi Kharrazi, Darrell J Gaskin, Suchi Saria

**Affiliations:** Department of Health Policy and Management, Johns Hopkins Bloomberg School of Public Health, Baltimore, Maryland, USA; Department of Computer Science, University of Virginia, Charlottesville, Virginia, USA; Department of Psychiatry and Behavioral Sciences, Johns Hopkins School of Medicine, Baltimore, Maryland, USA; Department of Computer Science and Statistics, Whiting School of Engineering, Johns Hopkins University, Baltimore, Maryland, USA; Department of Health Policy and Management, Johns Hopkins Bloomberg School of Public Health, Baltimore, Maryland, USA; Department of Health Policy and Management, Johns Hopkins Bloomberg School of Public Health, Baltimore, Maryland, USA; Department of Computer Science and Statistics, Whiting School of Engineering, Johns Hopkins University, Baltimore, Maryland, USA

**Keywords:** predictive model, hospital readmission, bias, health care disparity, clinical decision-making

## Abstract

**Objective:**

Health care providers increasingly rely upon predictive algorithms when making important treatment decisions, however, evidence indicates that these tools can lead to inequitable outcomes across racial and socio-economic groups. In this study, we introduce a bias evaluation checklist that allows model developers and health care providers a means to systematically appraise a model’s potential to introduce bias.

**Materials and Methods:**

Our methods include developing a bias evaluation checklist, a scoping literature review to identify 30-day hospital readmission prediction models, and assessing the selected models using the checklist.

**Results:**

We selected 4 models for evaluation: LACE, HOSPITAL, Johns Hopkins ACG, and HATRIX. Our assessment identified critical ways in which these algorithms can perpetuate health care inequalities. We found that LACE and HOSPITAL have the greatest potential for introducing bias, Johns Hopkins ACG has the most areas of uncertainty, and HATRIX has the fewest causes for concern.

**Discussion:**

Our approach gives model developers and health care providers a practical and systematic method for evaluating bias in predictive models. Traditional bias identification methods do not elucidate sources of bias and are thus insufficient for mitigation efforts. With our checklist, bias can be addressed and eliminated before a model is fully developed or deployed.

**Conclusion:**

The potential for algorithms to perpetuate biased outcomes is not isolated to readmission prediction models; rather, we believe our results have implications for predictive models across health care. We offer a systematic method for evaluating potential bias with sufficient flexibility to be utilized across models and applications.

## INTRODUCTION

The use of machine learning to diagnose disease,^1^^,^^2^ aid clinical decision support,^3^^,^^4^ and guide population health interventions^5^ has driven consequential changes in health care. While the data supporting the efficacy of these algorithms continues to mount, so too does the evidence that these models can perpetuate and introduce racial bias if not adequately evaluated.^6^^,^^7^ For example, a class of commercial risk-prediction tools that help health systems identify target patients for “high-risk care management” programs assigned the same level of risk to White patients and sicker Black patients. As a consequence of this bias, the number of Black patients identified for extra care was reduced by more than half.^6^ This inequality extends beyond hospital settings. Recently, the National Football League (NFL) was criticized for using race-based adjustments in dementia testing and for making it difficult for Black players to qualify for concussion claims.^8^ The NFL has since announced an end to the use of “race norming” when determining eligibility for concussion compensation; however, these examples reveal how pervasive medical racism remains.^8^

In response to these developing concerns, several reporting guidelines have been published to help researchers uncover potential issues in studies using prediction models.^9^^,^^10^ Researchers have also proposed mathematical definitions of bias,^11–13^ describing methods for measuring bias,^14–17^ and offering approaches for mitigating bias.^15^^,^^18^^,^^19^ While the development of these resources has been undoubtedly useful, they are limited in their comprehensiveness. For example, some frameworks assess only one element of algorithmic bias (eg, model training or optimization),^20–22^ while others only assess specific types of biases.^17^^,^^23^^,^^24^ By considering bias constituents in isolation, sources of inequality are likely to be missed. We refer to this effect—biases impacting algorithm performance across subgroups which leads to disparities from the algorithm’s use in the real world—as *disparate performance*.

The bias-related shortcomings of predictive models in health care are due, in part, to the failure to identify these concerns during algorithm design and reporting. If our ambition is to use machine learning to improve the health of patients irrespective of socio-economic status (SES) or race, fairness cannot be a fragmented or secondary consideration.^25^ The goal of our research was to develop a checklist with which model developers and health care providers can use to holistically assess an algorithm’s potential for disparate performance. By allowing these parties to appraise a model before it is deployed or even developed, potential for bias necessarily becomes a primary criterion of evaluation.

To evaluate our method, we applied the checklist to 4 of the most widely used 30-day hospital readmission prediction models. These models have been used to direct care to high-readmission-risk patients, standardize readmissions-based quality metrics across hospitals,^26^ and forecast all-cause and condition-specific readmissions.^26–28^ We selected this class of algorithms because of their prevalence^27–29^ and because reducing readmissions is a primary ambition for health systems and regulators.^30^

Moreover, there are established disparities in readmission rates in the United States—Black and Hispanic patients^31–34^ and patients with lower SES^35–38^ are known to have higher than average readmission rates. While these statistics do not inherently demonstrate bias, if readmission rates reflect disparities in the distribution of care, we must consider whether prediction models developed without accounting for these variations lead to disparate performance. To our knowledge, readmissions prediction research has only studied predictive performance, not disparate performance. We present the ways in which inter-group discrepancies can be introduced at each stage of the model development and deployment and how these differences have disproportionate effects on disadvantaged groups.

## MATERIALS AND METHODS

This study had 2 objectives: (1) develop a checklist that operationalizes the assessment of a model’s potential biases during model selection or before model deployment; and (2) assess if/how common 30-day readmission models might perpetuate health care disparities. The checklist was designed to surface possible biases and can thus guide supplementary quantitative assessments, mitigation efforts, and deployment considerations. When applied, the checklist questions uncover a model’s effect on both bias and disparity where we define bias as a difference in inter-group predictions, and disparity as a difference in health outcomes/quality due to disadvantaged attributes (eg, being of a specific racial group or having a low SES).^39^^,^^40^ Note that our definition of bias does not specify *how* inter-group predictions must differ (eg, algorithms may differ in terms of predictions made on otherwise identical patients, overall error rates, calibration, etc.). This is intentional as the bias of primary concern is contextually specific and we wish to consider a broad range of potential biases.

Our research methods included (1) our process for developing a bias screening checklist, (2) our process and criteria for identifying common 30-day hospital readmission prediction models, and (3) our process for assessing these predictive models using the checklist.

### Development of the bias evaluation checklist

We first gathered a team of experts in machine learning, health services research, health disparities, and informatics to develop a practical checklist for identifying potential biases in machine learning models. The checklist is a 3-step process: (1) understand background of the predictive task, which defines the disadvantaged groups and the types of biases and disparities of concern, (2) identify algorithm and validation evidence, and (3) use checklist questions to identify potential biases. The first 2 steps define objective of the predictive task and the parameters of deployment and step 3 is the in-depth assessment. The conceptual framework for the checklist was guided by several frameworks, including the 3 central axes framework,^41^ PROBAST,^9^ and the concepts of disparity and bias in Rathore 2004.^39^ We first separated the typical model development and deployment lifecycle into 4 phases: model definition and design; data acquisition and processing; validation; and deployment/model use. For each phase, we identified potential sources of bias, defined how each source could lead to bias and/or disparity, and established supporting examples. The potential sources of bias and their mechanisms were summarized through synthesizing literature and discussion with multidisciplinary stakeholders whose work relates directly to 1 of the 4 phases. Lastly, we created guiding questions to help those applying the checklist identify these potential sources of bias. The questions were developed based on extensive literature review and expert opinions. The checklist was refined iteratively through working sessions and pilot tests.

### Selection of algorithms for analysis

To select algorithms for analysis, we performed a literature search *in the PubMed*, *Embase*, and *Google Scholar databases* to identify all-cause 30-day hospital readmission prediction models and their corresponding validation or comparison studies. Our review started with the assessment of the readmission models covered in several systematic reviews.^26–29^^,^^42^ An additional search was conducted for 30-day readmission models published after June 2019 as models developed after this date were not covered by the systematic reviews.

To be included in our assessment, algorithms had to predict 30-day hospital readmissions at the patient-level and must have been based on claims data or electronic health records (EHRs). All model types (eg, linear models, deep learning) were considered. Models that predicted readmissions for specific conditions (eg, patients with congestive heart failure), or that used risk factors not typically available in EHRs, discharge records, or insurance claims (eg, living arrangement, frailty assessment) were excluded. We also excluded studies that did not establish a predictive model (eg, determined the association between a certain risk factor and readmissions).

We prioritized assessing commonly used models. To qualify as “common,” an algorithm must have been validated, evaluated, or applied in 2 or more external settings. To determine if a model met our definition of common, we conducted a literature search to identify external validation studies and comparison studies for each model that met our inclusion criteria.

**Figure 1.**
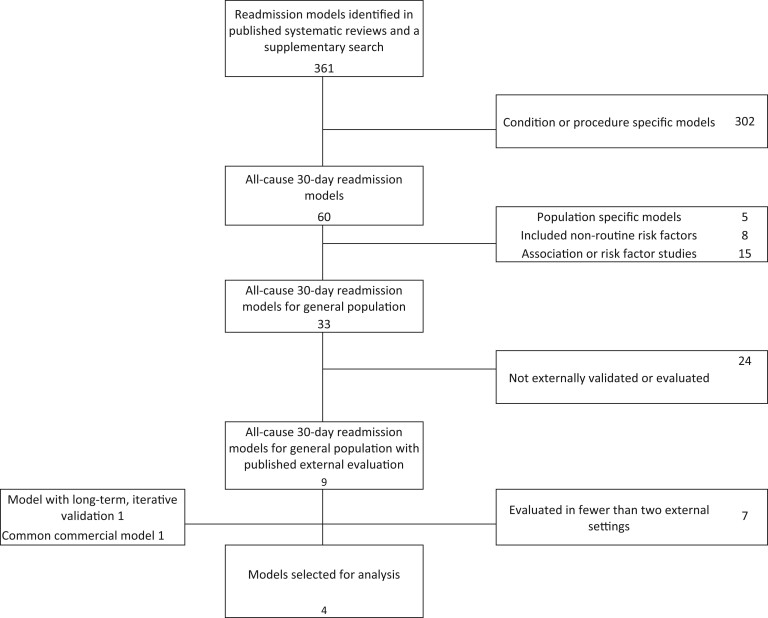
The PRISMA diagram for selecting common 30-day hospital readmission models.

After applying these inclusion criteria, we were left with 2 of the most well-studied 30-day readmission models—LACE and HOSPITAL.^43–53^ To broaden our analysis, we also chose to assess HATRIX^54^ and the readmission model in the Johns Hopkins ACG system.^55^ We selected HATRIX because its validation study was conducted iteratively over 2.5 years. The length of this analysis means HATRIX provided rare insights into temporal effects on model validity.^54^^,^^56^ The Johns Hopkins ACG system is one of the most widely applied commercial risk adjustment tools. The system’s broad commercial use, the international validation of ACG’s utilization and health care needs predictions,^57–61^ and the relative availability of its documentation warranted the model’s inclusion. The review process is illustrated in Figure 1.

### Analyzing bias in the selected algorithms

Lastly, we evaluated the common 30-day readmission models using our checklist. Each model was assessed by 1 researcher and verified by at least 2 others to ensure consistency across all judgments and descriptions. Disagreements and comments were resolved during working sessions wherein the research team reviewed evidence, evaluated intent, consulted experts if needed, and ultimately defined an answer for the question under consideration.

## RESULTS

Our checklist gives model developers and health care providers a means to systematically assess an algorithm’s potential for disparate performance across subgroups. The checklist consists of 3 steps. First, a user must clearly define what the model predicts and how it should be used. Second, a user should find evidence of the algorithm’s efficacy. Third, a user must answer 11 guiding questions to identify 6 sources of potential bias in step 3 (Table 1). These questions are organized into 4 stages, one for each step of model development.

**Table 1.** Bias evaluation checklist to assess the potential for a machine learning model to introduce bias and perpetuate disparate performance across subgroups

Source of bias	How the bias can arise	Example(s)	Checklist question(s)
Stage 1: model definition and design			
Label bias	The use of a biased proxy target variable in place of the ideal prediction target during model learning.	Health systems often rely on prediction algorithms to identify patients for their “high-risk care management” programs. The ideal prediction target for these models is patients’ future health care needs, and algorithms often predict the value of a concrete proxy variable—future health care costs to represent patient’s future health needs. Black patients typically have lower health care costs as they are less likely to seek or receive care. Consequently, algorithms that predict future health care costs as a surrogate for future health care needs create disparities in medical decision-making for tens of millions of patients.^62^	Is the prediction target an appropriate proxy for patient health care outcomes or needs?
Modeling bias	The use of a model that, due to its model design, leads to inequitable outcomes.	One study found colon cancer screening, sinusitis, and accidental injury to be statistically significant predictors in a stroke risk prediction model. However, these data are not actually relevant to stroke prediction. Instead, they simply represent high utilization of health care resources. Using these data can therefore create performance disparities between patients with low health care utilization and those with high health care utilization.^63^ Blacks and socioeconomically disadvantaged groups have poorer access to care and lower health care utilization. Consequently, these groups could be adversely impacted by such a model. —————— Lending algorithms sometimes make decisions from nonuniversal generalizations—such as the neighborhood in which an applicant lives—instead of applicant-specific data. By using neighborhood-level data and excluding important individual-level inputs, lending models cannot capture the variation within each subpopulation that would result in different outcomes for different individuals. As a result, qualified applicants that live in disqualified neighborhoods are denied loans without merit.^64^^,^^65^	Are there any modeling choices made that could lead to bias? For example, are there any dependencies between inputs and outcomes that could lead to discriminatory performance across groups? Are any important features excluded from the model? Does the model algorithmically account for bias? For example, does the model attempt to limit bias as part of its optimization criteria? Does the model account for training data imbalance?
Stage 2: data collection and acquisition			
Population bias	The algorithm performs poorly in subsets of the deployment population because the data used for model training does not adequately represent the population in which the algorithm will operate.	A melanoma detection model achieved accuracy parity with a board-certified dermatologist; however, the model was trained primarily on light-colored skin. As such, the algorithm is likely to underperform for patients with dark skin. The potential benefit of early detection through machine learning will thus be limited for these patients.^1^ —————— Amazon used an algorithm to review job applicants’ resumes. However, the model favored male candidates because it was trained with data from a period during which most applicants were men.^66^	Was the data used to train the model representative of the population in the deployment environment? If not, was the model developed to be robust to changes in the population?
Measurement bias	Bias introduced because of differences in the quality or way in which features are selected and calculated across different subgroups.	Under-served subgroups are disproportionately assessed as high-risk borrowers and are thus less likely to have their mortgages approved. The difference in mortgage approval rates is due to the relative absence of data (eg, short credit history, non-diverse in types of loans) for minority groups. As a result of missing data, prediction algorithms are less precise for minorities which leads to the approval rate inequity.^67^ —————— Machine learning algorithms typically require large datasets for training. Existing biomedical datasets have historically misrepresented or excluded data for immigrants, minorities, and socioeconomically disadvantaged groups.^68^ As a result of the misrepresentation of their data, these groups can suffer adverse health outcomes, such as incorrect diagnosis.^69^ Simulations demonstrated that including even small numbers of Black Americans in control cohorts likely would have prevented these misclassifications.^69^	Are input variables defined and measured in the same way for all patients? Was the prediction target measured similarly across subgroups and environments? Are input variables more likely to be missing in one subgroup than another?
Stage 3: Validation			
Missing validation bias	An absence of validation studies that measure and address performance differences across subgroups.	Machine learning models are often not assessed for disparate performance across subgroups before they are deployed. This has led to the introduction and perpetuation of bias in kidney transplant list placement,^70^^,^^71^ criminal sentencing,^72^ facial recognition systems,^73^ and other consequential applications.^74^ —————— The external validation of an acute kidney injury prediction model with excellent performance at the source hospital demonstrated deteriorating performance at 5 external sites due to the heterogeneity of risk factors across populations.^75^	Do validation studies report and address performance differences between groups?
Stage 4: Deployment and model use			
Human use bias	An inconsistent response to the algorithm’s output for different subgroups by the humans evaluating the output.	In a study to assess the effect of criminal justice risk prediction algorithms, judges were presented with vignettes that described a defendant’s index offense, criminal history, and social background. Some judges were also provided with a defendant’s estimated likelihood of re-offending. For affluent defendants, the probability of incarceration decreased from 59.5% to 44.4% when risk assessment information was provided. For relatively poor defendants, the addition of risk assessment information increased the probability of incarceration from 45.8% to 61.2%. Thus, the authors concluded that, in some cases, providing judges with risk assessment scores can exacerbate disparities in incarceration for disadvantaged defendants.^76^ —————— A machine learning algorithm developed to help pathologists differentiate liver cancer types did not improve every pathologist’s accuracy despite the model’s high rate of correct classification. Instead, pathologists’ accuracy was improved when the model’s prediction was correct but decreased when the model’s prediction was incorrect. This demonstrates the potential unintended effects of using an algorithm to guide decision-making.^2^	Might a user interpret the model’s output differently for different subgroups? Might the use of the model perpetuate disparities even if the model’s predictions are accurate across groups? Might the model’s output lead to more uncertainty in decision-making (eg, if the model’s output is ambiguous)?

We evaluated LACE, HOSPITAL, HATRIX, and Johns Hopkins ACG with our checklist. All 4 are logistic regression models that predict a patient’s risk of being readmitted to a hospital within 30 days of discharge based on clinical characteristics and health care utilization history. The results of this analysis are summarized in this section. The unabridged results are included in Supplementary Appendix 1.

### Step 1: defining how the model will be used

We defined our operational setting as a hypothetical hospital system that is seeking to reduce readmission rates. To most appropriately manage the discharge and post-acute care follow-up for patients at high risk of unplanned readmission, this hospital employs an algorithm to predict which patients are most likely to be readmitted. In regard to bias, the hospital is most concerned with the inequitable treatment of Blacks and those with low SES given the evidence of higher readmission rates for these groups.^31–34^^,^^36^^,^^38^

### Step 2: compiling and examining prior evidence for each algorithm

The respective external validations studies for LACE, HOSPITAL, HATRIX, and Johns Hopkins ACG measured performance for different populations (eg, hospital system or country).^44–46^^,^^48^^,^^49^^,^^51–53^^,^^56^^,^^57^^,^^59^^,^^60^ However, no studies examined disparate performance for the relevant subgroups (ie, performance for Black patients relative to White patients).

### Step 3: identifying and evaluating potential sources of bias

Our checklist allows users to uncover potential sources of bias, consider the magnitude of each bias’s effect on disparate performance, and rate the level of concern for each type of bias. By design, the checklist questions are grouped by model development stage.

#### Model development stage 1: definition and design

We found each model’s prediction target to be potentially concerning. LACE, HOSPITAL, and Johns Hopkins ACG predict unplanned readmissions, while HATRIX predicts global readmissions. Both unplanned and global readmissions are measures of health care utilization, not health care needs. Hospital utilization is driven by insurance coverage and access, willingness to seek care, the resources of local hospitals, and racially associated social conditions.^77^^,^^78^ More utilization only means a patient uses more health care resources; it does not necessarily mean that a patient requires more care. In this way, health care utilization is an inadequate proxy for health care needs. Thus, using readmissions to represent underlying health care needs could lead to the systemic underestimation of risk for those with higher barriers to access care.

We also found concerns related to each model’s design. All 4 algorithms depend on routinely collected data including health care utilization history, lab tests, and medications. These data can lead to biased health care outcomes. For example, Black and low SES patients are more likely to visit the Emergency Department (ED) for routine care and non-urgent reasons.^79^ The difference in number and severity of ED visits may affect a model’s analysis of risk across groups. Moreover, each model relies on diagnoses, clinical severity, and comorbidities. These data are subject to different practice and coding intensity (eg, frequency of diagnoses).^80–82^ Therefore, using these data can adversely affect those who lack access and visit health systems with lower practice intensity.

Finally, LACE and HOSPITAL rely on relatively few inputs (4 and 7, respectively). While simplicity can be attractive, missing important features can have a profound effect on readmission prediction. For example, one study demonstrated the differential readmission rates for myocardial infarction patients across races disappeared after adjusting for a comprehensive set of patient factors.^83^ If a model does not account for these factors, its use may lead to biased health outcomes.

#### Model development stage 2: data collection and acquisition

We found concerns related to the difference in the data used for model training and the data used for making real-world predictions. For example, the Johns Hopkins ACG models were developed with claims data; however, many hospitals feed EHR data to their deployed ACG models. This is problematic because some data may not be identically represented across these 2 data sources. Consider medication prescriptions. When a doctor prescribes a drug, the event is invariably represented in an EHR while claims data only captures filled prescriptions.^83^^,^^84^ Patients may not fill a prescription for several reasons including expense, concerns about the medication, lack of perceived need, lack of trust with the provider, or lack of access.^85^ Since Blacks have a lower prescription fill rate and medication adherence than Whites,^86^^,^^87^ it is possible that using EHR data in a model developed with claims data (or vice versa) could lead to disparate performance across subgroups.^88^

Our checklist also identified concerns regarding the lack of a standard definition for an “unplanned readmission.” There are several approaches that can be used to determine whether a readmission is planned or unplanned including patient interviews,^47^ the SQLape algorithm,^89^ and the CMS methodology.^90^ When a model’s definition of unplanned readmission does not match the health system’s, adjustments are often made to suit the local context. For example, some institutions use hospitalizations resulting from an ED visit as a proxy for unplanned admissions. No research has assessed how these adjustments impact different subgroups’ readmissions rates.

For each model, we also found the potential for bias to arise from different rates of data availability and data quality across subgroups. Health care utilization history is a key predictor in the models we analyzed. Certain subpopulations (eg, those with housing challenges, unstable employment, or lack of insurance coverage) are more likely to have fractured or lower-quality care and more limited access to care.^91^^,^^92^ In these cases, hospitals must join disparate data sources to form a complete account of a patient’s history—a task that is often impractical if not impossible. Additionally, patients with lower health literacy may not be able to report all their health events or may lack access to the online patient portals in which care received at other institutions is recorded.^92^

We also found each model’s use of test results and medications to be problematic. Because race and SES can affect the treatment a patient receives, access to diagnostic tests, and the number of diagnostic tests conducted,^93^^,^^94^ these data may cause prediction algorithms to unduly assign higher risk to patients with greater access to care.

#### Model development stage 3: validation

Despite the popularity of these models, there are no studies that assess the disparate impact of LACE, HOSPITAL, HATRIX, or ACG across racial or SES groups. To our knowledge, the only related research evaluated 50 prediction tasks using embeddings from medical notes.^95^ The authors concluded that predictive performance favored the majority group; thus, we cannot rule out the potential for performance disparities across subgroups.

#### Model development stage 4: deployment and use

Even if a model is completely free of bias, there is potential for inequality to arise from a user’s response to a model’s output. LACE, HOSPITAL, and HATRIX generate a score to represent readmission risk. Practically, this means users must define a threshold above which a “high risk” intervention is triggered. For example, patients with LACE scores above 10 are typically considered high risk, however, evidence to support this threshold is mixed.^51^^,^^96^^,^^97^ It is unclear how different “high risk” thresholds might affect health outcomes across subgroups.

To our knowledge, there is no literature reporting the impact of LACE, HOSPITAL, HATRIX, or ACG on clinical decision-making. However, available evidence demonstrates that prediction scores account for only a part of a provider’s perception about a patient’s readmission risk.^98^ In fact, for one readmission prediction algorithm, the score and the readmission prevention program enrollees were congruent in only 65% of patients.^99^ These findings are valuable; however, without additional evidence, we cannot draw conclusions about the effect of readmission prediction algorithms on disparate performance.

**Figure 2.**
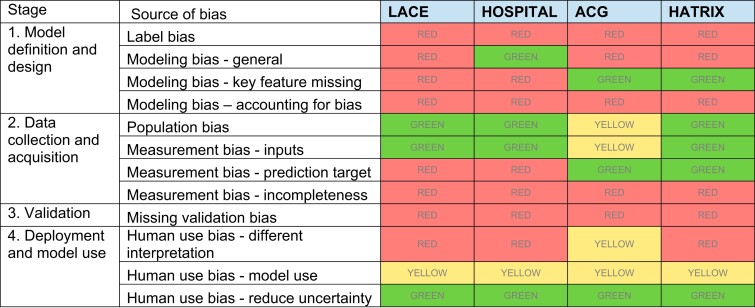
Model assessment heat map. An overall rating was given for each bias type based on the qualitative assessment of the checklist questions (details in Appendix 1). Red indicates there is potential for concern, green indicates there is limited potential for concern, and yellow indicates the potential for concern is unclear or there is not enough information with which to draw a conclusion.

Overall, our results demonstrate that LACE and HOSPITAL introduce the most areas of possible bias, Johns Hopkins ACG has the most sources of uncertainty, and HATRIX has the fewest causes for concerns. Importantly, this does not mean any one of these models is inherently better or worse than the others. Rather, our results indicate the areas that must be most thoroughly assessed by health systems intending to use one of these models. The summary is illustrated in Figure 2.

## DISCUSSION

We have developed a practical and systematic method for uncovering the ways in which a machine learning model can perpetuate bias in health care. To assess our proposed approach, we applied our checklist to 4 common 30-day readmission risk prediction models—LACE, HOSPITAL, HATRIX, and Johns Hopkins ACG. Despite being widely deployed and available for more than a decade, these models have undergone limited or no bias-related evaluations. This is particularly concerning given our checklist exposed several ways in which these algorithms can lead to disparate performance across subgroups. The sources of bias we identified are not unique to readmission models—they can arise in nearly any health care prediction algorithm, many of which are far more complex than the readmission prediction models we assessed. While our analysis focused primarily on race and SES due to the evidence of disparities in readmission rates across these groups,^31–34^^,^^36^^,^^38^ other types of demographic biases are equally important and likely to arise across other areas of healthcare.^100^ Although the algorithms analyzed in this article are relatively straightforward logistic regression models, it remains important to assess whether these models can be deployed to new settings with equitable impact to various subpopulations, and what factors may hinder the models’ generalizability (eg, distribution shifts, temporal effects etc.).^101–103^

Generally, the assessment of an algorithm’s bias has been reduced to statistical testing of performance across subgroups.^12^^,^^14^^,^^15^^,^^17^^,^^104^ Our results illustrate the necessity for new bias evaluation and management tools that allow model developers and health care providers to understand the sources, impact, and mechanisms of disparity. For example, we found routine EHR and claims data—such as utilization history, diagnoses, and procedures—are subject to racial differences in completeness and quality. While it is clear models relying on these data can lead to biased health care outcomes, the reasons for and magnitude of the disparity cannot be determined using quantitative methods because the “truth” is often unavailable. For this reason, a qualitative approach can be more effective at identifying sources of bias—a task critical to predicting how a model may lead to disparities in an operational setting.

Traditional bias assessment methods are also unable to evaluate how users interpret and act based upon a model’s output. This relationship is notoriously difficult to evaluate; however, it is important to consider given its direct impact on health outcomes and because the interaction between a model and health care provider are often not systematic. In fact, a recent review on automation bias identified a wide range of user and environmental factors that affect a user’s reliance on a model’s output.^105^ For example, it is not uncommon for risk thresholds to be defined to maximize the benefit of an intervention given resource constraints after a model is deployed, not by some consistent method.^106^ A user’s interaction with a model can also be complicated by its transparency and interpretability. For example, clinicians may struggle to trust the algorism due to large number of inputs and the difficulty to explain the logic behind an alert,^107^ but they also showed willingness to trust the algorism if they understand how the system works in different scenarios.^108^ In practice, the cooperation between a human decision-maker and an algorithm adds layers of complexity to the potential for biased outcomes. Thus, this interaction must be considered with the same scrutiny as every other stage in the model’s development and deployment.

Our checklist addresses each of these concerns by allowing model developers and health care providers elucidate how bias might arise at each phase of an algorithm’s development, deployment, and use. Because bias can arise from the data, model, workflow, or the intervention design, a multidisciplinary team (data scientists, statisticians, clinicians, informaticians, etc.) is required to comprehensively identify bias and devise appropriate mitigation methods.^109^ For example, a machine learning scientist may employ feature selection techniques to optimize a model, however, a health practitioner or clinician must assess whether the selected features make sense given established knowledge and whether the algorithm may have eliminated features that are relevant for potential algorithmic bias. Given our analysis demonstrates that the early phases of model development—such as defining a prediction objective and selecting data sources—are particularly prone to introducing bias, these efforts should begin as early as possible.^25^Definitions of bias and fair practices have been increasingly scrutinized as machine learning models have proliferated in health care. For example, there has been a rich debate regarding the use and impact of sensitive data such as race as inputs to any predictive algorithm.^110–113^ These considerations have extended beyond pure performance to issues such as privacy.^114^ We believe these discussions are critical and should be had within the context of a specific algorithm and use case. The inclusion of sensitive data should be based on the potential for latent discrimination even in the absence of sensitive data, the relative availability and completeness of sensitive attributes, a priori knowledge of which sensitive features are responsible for bias, and many other related factors.^112^^,^^113^ Uniformly defining which features should or should not be included in a model is overly restrictive. Our checklist was designed to give model developers a framework with which to discuss these sensitive yet important topics.

This study had a few limitations and caveats. First, we assessed the readmission prediction models in the context of a hypothetical health system, thus we had to simplify several practical matters. Additionally, without quantitatively assessing each models’ performance, we were unable to precisely identify the magnitude of subgroup disparities or make definitive conclusions about each model’s fairness. Moreover, since our assessment was based on published literature, our findings largely depend on the quantity and quality of the reporting. Finally, our qualitative assessment may not be sufficient to propose mitigation or model design strategies. Future research should define the methods best suited to prevent or limit specific disparities across vulnerable population groups.

## CONCLUSION

Despite the enthusiasm surrounding the use of algorithms to guide clinical and population health interventions, a growing body of evidence indicates that these tools can lead to inequitable outcomes across racial and socio-economic groups. Biased results are problematic, however, the absence of methods for systematically evaluating the models that produce these outcomes is even more concerning. In effect, sophisticated yet opaque tools are being used to make consequential health care recommendations, yet we have few methods to assess their racially disparate consequences. The checklist we introduce allows model developers and health care providers to systematically assess a model’s potential to introduce bias. Because reducing hospital readmissions is a notable initiative for health care providers and policy makers, we evaluated our method by assessing 4 of the most widely deployed 30-day readmission prediction models. Our results demonstrate that, despite the significant effort applied to the development of readmission prediction algorithms, there are several critical ways in which these models can perpetuate growing health care inequalities. While we assessed readmission models, our framework was designed to be flexible such that it can be used to evaluate bias in other health care domains and applications.

## FUNDING

This research received no specific grant from any funding agency in the public, commercial or not-for-profit sectors.

## AUTHOR CONTRIBUTIONS

HEW, HK and SS conceived the study concept. HEW, ML, RA, AS, SS developed the conceptual framework and checklist, and participated in working sessions to reach consensus on checklist and assessment results. HEW conducted the scoping review. HEW and ML analyzed the data, conducted the pilot assessment and wrote the manuscript. All authors reviewed and discussed the checklist design, assessment results and contributed to the final manuscript.

## SUPPLEMENTARY MATERIAL

Supplementary material is available at *Journal of the American Medical Informatics Association* online.

## CONFLICT OF INTEREST STATEMENT

HEW is an employee of Merck. Co and the employer had no role in the development or funding of this work.

## DATA AVAILABILITY STATEMENT

All data are incorporated into the article and its online supplementary material.

## Supplementary Material

ocac065_supplementary_data
