# Correction: A bias evaluation checklist for predictive models and its pilot application for 30-day hospital readmission models

**DOI:** 10.1093/jamia/ocac102

**Published:** 2022-06-17

This is a correction to: H Echo Wang, Matthew Landers, Roy Adams, Adarsh Subbaswamy, Hadi Kharrazi, Darrell J Gaskin, Suchi Saria, A bias evaluation checklist for predictive models and its pilot application for 30-day hospital readmission models, *Journal of the American Medical Informatics Association*, 2022; ocac065, https://doi.org/10.1093/jamia/ocac065

In the originally published version of this manuscript, the first author’s name includes an extra word “Echo”, the first author’s name should be “H. Echo Wang”

This error has been corrected (online).

