# Supplementary material for: A bias evaluation checklist for predictive models and its pilot application for 30-day hospital readmission models
Source: J Am Med Inform Assoc. 2022 May 17;29(8):1323–33. doi: 10.1093/jamia/ocac065 (PMC9277650; doi:10.1093/jamia/ocac065)
Supplement: ocac065_supplementary_data [file ocac065_supplementary_data.docx]

Appendix 1: Detailed results of evaluation of potential bias in common 30-day readmission predictive models

| **Section 1. Describe the purpose of the predictive task and the deployment or operational setting.** | |
| --- | --- |
| 1.a) What is the intended use --- identify setting, user, how it will be used (allocation), outcomes it will impact, and data available? | **Goal**: Identify patients at high risk of unplanned/avoidable 30-day readmission and provide post-acute follow up care in order to mitigate 30-day readmissions **Setting**: At or near time of discharge within an encounter in a hospital **Prediction Target**: Readmission within 30-days of discharge **User**: Clinicians or discharge planners **Data available**: Physiology, Comorbidities, Clinical History, Demographic, Medications, Procedures, Provider Information, Certain Social factors **End user**: Case managers, nurses, or clinicians who are discharging patients |
| 1.b) What are the subgroups at risk for disparity in the target population (relevant groups)? | Low income/SES, and non-White (racial minority) |
| 1.c) Are there group differences in the prediction target (e.g. 30-day readmission rates are higher among black patients than white ones)? | Evidence of readmission disparity for blacks, low-income/SES, those with limited English proficiency. |
| 1.d) What types of disparities are concerning in the intended setting? | The following types of disparities are particularly concerning:  (a) unequal allocation of intervention resources (certain subgroups fail to receive adequate post-acute follow up care because the predicted risk is underestimated) (b) unequal outcome as a result of inadequate access to interventions necessary for mitigating 30-day readmissions. |
| 1.e) What types of performance disparities could lead to inequitable resource allocation? | If the risk score systematically under- or over- estimate patient outcome/needs for a certain group, it would lead to inequitable resource allocation that is unfair to the disadvantaged group. For example, if the algorithm has a much higher false negative rate for a disadvantaged group, it would be problematic as the disadvantaged group would end up receiving less resource than it should have. |

| **Section 2. Describe the algorithms being considered and validation evidence** | | | | |
| --- | --- | --- | --- | --- |
|  | **LACE Index** | **HOSPITAL Score** | **Johns Hopkins ACG** | **HATRIX** |
| 2.a) What was it developed to do and on what data? | LACE was developed to predict risk of unplanned readmission or death after discharge from the hospital, to help clinicians identify patients who might benefit from more intensive post-discharge care. It was developed in a multicenter prospective cohort study in Ottawa, Canada. | Developed as a prediction model for potentially avoidable 30-day hospital re- admissions in medical patients using administrative and clinical data readily available prior to discharge. | Hopkins ACG is a suite of tools to identify patients at high risk, forecast health care utilization and set equitable payment rates. Healthcare organizations usually re-train or calibrate the model locally before deployment. It was developed using IMS claims data, which includes ambulatory data and medications. The model of interest is its unplanned readmission risk prediction model. | HATRIX was developed to predict a patient’s likelihood of readmission, utilizing a health systems electronic medical record. It was developed through a retrospectve data extraction and analysis study in a multi-site academic medical center in Detroit, MI. It was integrated into the EMR. |
| 2.b) What validation studies exist and what do they report? | It was validated & evaluated internationally (e.g. UK, France, Singapore, Denmark, CA, US) in different settings (e.g. elderly, general medicine, heart failure. community hospital, inpatient). The accuracy is moderate in general (C statistic is between 0.58 and 0.65) | This retrospective cohort study included consecutive adult patient discharges from all medical services of the Brigham and Wo- men’s Hospital with a discharge date between July 1, 2009, and June 30, 2010. Had fair discriminatory power (C statistic, 0.71) and had good calibration. | Validation studies on ACG's readmission model is limited but validation for other ACG models (e.g. hospitalization, expenditure, mortality) show good validity. In addition, ACG groups are widely used in models predicting other healthcare outcomes. | Validation cohort shows AUC =0.72. An internal iterative validation process was published to assess 9 internal validations in clinical practice over 2.5 years. |

| **Section 3. Identify potential sources of bias in implementing a particular model.** | | | | |
| --- | --- | --- | --- | --- |
| **3.a) Bias related to model definitions and design** | | | | |
|  | **LACE Index** | **HOSPITAL Score** | **Johns Hopkins ACG** | **HATRIX** |
| i) Is the prediction target an appropriate proxy for patient healthcare outcomes or needs? | Could be a concern  Hospital inpatient readmission risk, especially avoidable/unplanned readmission risk is an appropriate proxy for adverse patient outcome, unnecessary patient/health system burden, and the need for discharge management.  The original model defined a readmission as unplanned if they had not been arranged or planned at discharge and it was ascertained by patient interview at discharge. The method heavily depends on patient’s ability and willingness to access care, and could have embedded bias toward disadvantaged groups. | Could be a concern The potentially avoidable readmission in original setting is considered to be unavoidable if (1) planned readmission (e.g., scheduled at the time of the index admission, planned treatment follow-up, and planned chemotherapy) or (2) unforeseen readmission for newly developed conditions not related to known diseases during the index hospitalization. It is  determined using SQLape algorithm. There is no evidence that suggests this algorithm has validity issue and the author also manually excluded some planned readmissions. However, the SQLape algorithm requires additional clinical information to calculate the outcome, which could reduce sample size or underestimate the outcome for disadvantaged groups. | Could be a concern In model development, unplanned readmission was identified using the methodology developed by CMS (Horwitz 2015). The CMS methodology is well validated and updated annually to reflect the revisions in ICD coding, and no evidence shows the unplanned readmission methodology has any validity or disparity issues. However, the methodology requires additional clinical information to calculate the outcome, which could reduce sample size or underestimate the outcome for disadvantaged groups. | Could be a concern The prediction target in the original setting is all-cause 30-day readmission (did not distinguish between planned or unplanned). The determination of prediction target does not rely on any additional methodology and no indication of bias. However, the overall readmission is not as accurate as unplanned readmission for predicting adverse health outcome and needs, and the effect on subgroups is unclear. |
| ii) Are there any modeling choices made that could lead to bias? For example, are there any dependences between input and outcome that could lead to discriminatory performance across groups? | Could be a concern  Among the four input variables of LACE index, number of ED visits in the previous 6 months is likely to differ between racial and SES groups and could lead to differential scores mainly due to race/SES. Research shows blacks and Hispanic groups are more likely to visit ED for non-urgent conditions, potentially because they are more likely to rely on ED for routine care (Zhang 2020). | Not a concern Among the 7 input variables of HOSPITAL score, none of the variables have established association to underlying discriminatory factors. | Could be a concern The input variables are very comprehensive (considered all comorbidity, inpatient and ED utilization and age) and the modeling choice is reasonable. Although ACG groupings are well validated, it is unclear if generating these groupings require high level of record completeness. Additionally, these input variables are subject to coding intensity & patient’s access to care, which could vary across race and SES groups. | Could be a concern The hospital admission history only includes admissions to the study hospital system and would not include those had prior hospitalization elsewhere. Blacks and low SES are shown to be more likely to have fragmented care and the variable could underestimate the prior hospitalization of disadvantaged groups. |
| iii) Are any important features excluded from the model? | Could be a concern With only 4 predictors, it is possible key features are not included in the model, such as diagnoses that are likely to have readmissions. Moreover, area-level data (neighborhood, SES, etc.) were specifically and explicitly not included so the extent that these features are predictive of the outcome, LACE will not account for them | Could be a concern The risk is similar to LACE. In addition, the model author acknowledged the algorithm did not include important predictors such as functional status, health literacy, social support and medication adherence, which are often difficult to obtain. | Not a concern  The feature selection process has reasonable consideration to include a comprehensive list of features and the algorithm is widely used in different populations and refined over time. | Not a concern  Given the variables were carefully down-selected to 10 and the iterative validation has captured all relevant variables, the probability of missing key features is low.  Length of stay was not in the original model, but a later publication assessed LOS in its iterative validation process and added it to the model |
| (iv) Does the model algorithmically account for bias? For example, does the model attempt to limit bias as part of its optimization criteria? Does the model account for training data imbalance? | Could be a concern  No specific process to limit bias or account for imbalance. The original article did not report race or SES distribution thus unable to infer it there are enough samples for subpopulations given readmission is a relatively rare outcome. | Could be a concern  No specific process to limit bias. The training data has 16.3% black and 8% Hispanic and readmission cases are low in these two subgroups. | Unclear  The original readmission model had no specific process to limit bias. There is no report of demographic distribution of the training data (only know it is based on IMS claims, which representing privately insured patients). | Could be a concern  No specific process to limit bias. The training data is predominantly black (66% - 75.5%), followed by white; the sample sizes and outcome cases of these two groups seem reasonable. |

| **3.b) Bias related to data collection and acquisition** | | | | |
| --- | --- | --- | --- | --- |
|  | **LACE Index** | **HOSPITAL Score** | **Johns Hopkins ACG** | **HATRIX** |
| i) Was the data used to train the model representative of the population in the deployment environment? If not, was the model developed to be robust to changes in the population? | Not a concern  The model was originally trained based on administrative data and perspective interviews from patients in 11 hospitals in Ontario. The score-based index was validated in various hospital populations. It is appropriate to use for general inpatient population in a hospital. | Not a concern  The model was trained on EHRs data from patients from medical services in a large academic hospital in US. It is appropriate to use for general inpatient population in a hospital. | Could be a concern in EHRs  The model was trained on claims data and is appropriate for another health plan and would require retraining and/or other adjustments if using EHRs data or hospital setting. | Not a concern  The model was trained on EHRs data from an academic hospital system (8 hospitals) in US. It is appropriate to use for general inpatient population in a hospital. |
| ii) Are input variables measured and defined in the same way for all patients? | Not a concern Inputs:  - Length of stay - Admission type as Acute or Chronic - Charlson Comorbidity Index (CCI) score - Emergency department visits in last 6 months  The risk of differential measurement and definition of these input variables are low. | Not a concern Inputs: hemoglobin at discharge, discharge from an oncology service, sodium level at discharge, procedure during the index admission, index type of admission, number of admissions during the last 12 months, and length of stay There is no evidence to suggest that these variables would be measured differently across subgroups | Maybe a concern in EHR  Since the model was trained in claims database, some input variables may not be available or measured the same in EHRs, such as medications, and ED history. | Not a concern Input: 10 variables including previous admissions in last 12 months, diagnoses, medications. The risk of differential measurement and definition of these input variables are low. |
| iv) Was the prediction target measured similarly across subgroups and environments? | Could be concern The unplanned readmission was determined using pre-discharge interview during training. It would not be the same in the deployment environment.  Its external validation required stitching together three different databases (will not be the same in deployment setting either).  Some subgroups could be more likely to be missing data. Using aggregated DBs to form a complete picture of a patient's admissions/ readmissions may further disadvantages the groups for which data is more likely to be missing. | Could be a concern The 30-day unpanned readmission is the prediction target, determined using SQLape and some manual adjustments, and seems to be defined and measured the same way for all.  The target would not be the same in deployment setting unless it also subscribes to SQLape. | Not a concern The unplanned 30-day acute care hospital readmission is defined using CMS methodology and measured the same way for all. The target would be measured the same way in deployment setting. | Not a concern The all-cause 30-day readmission is prediction target and seems to be defined and measured the same way for all. The target would be measured the same way in deployment setting. |
| v) Are input variables more likely to be missing in one subgroup than another? | Some evidence (Could be concern) Evidence of condition-specific disparities in diagnoses or recording of comorbidities  ED visit history may be more complete in some group than the other | Some evidence (Could be concern)  If patients seek care in different hospital system, the documentation of previous admissions would likely to incomplete. Disadvantaged groups are more likely to have fractured care. These input variables may not capture complete data for those with fragmented care (e.g. # of admission in past 6 months) and the limited # of features may not account for variations affected by race. | Some evidence (Could be concern)  Those with more fragmented care are likely to be missing inpatient and ED history if they receive care elsewhere; EHR system only has medication prescribed, and those are more economically disadvantaged or have poorer access to medications would not have accurate representation. | Some evidence (Could be concern) If patients seek care in different hospital system, the documentation of previous admissions would likely to incomplete. Disadvantaged groups are more likely to have fractured care. |

| **3.c) Bias related to model validation** | | | | |
| --- | --- | --- | --- | --- |
|  | **LACE Index** | **HOSPITAL Score** | **Johns Hopkins ACG** | **HATRIX** |
| i) Do validation studies report and address performance differences between groups? | No evidence (Could be a concern) Validation on LACE has been conducted in many populations and health system but none has evaluated racial or SES differences.  Found at least 7 different validation studies. Measured c-statistic for different subpopulations (e.g., by procedure, condition, age) but not race. | No evidence (Could be a concern) Validation studies did not rule out such group differences. | No evidence (Could be a concern)  Validation studies did not rule out such group differences. | No evidence (Could be a concern) Validation studies did not rule out such group differences. |

| **3.d) Bias related to deployment and model use** | | | | |
| --- | --- | --- | --- | --- |
|  | **LACE Index** | **HOSPITAL Score** | **Johns Hopkins ACG** | **HATRIX** |
| i) Might a user interpret the model’s output differently for different subgroups? | Could be a concern with varying threshold. User will need to determine the score threshold for "high risk." Most studies have been using 10, or other higher scores in different settings. One threshold may not indicate the same risk in different subgroups and it is unclear how varying threshold affects the classification disparity. | Could be a concern   HOSPITAL evaluates patients as either high, medium, or low risk for readmission. The % of patients expected to get readmitted vary based on risk threshold. It could be an arbitrary decision to act on high vs. high& medium risk patients. It is unclear if user responds differently to these risk levels for different subgroups, however if a user did respond differently for different subgroups this would lead to inequitable allocation of resources. | Unclear  ACG is designed to be integrated into the EHR or case management system to inform intervention. The dashboard displays multiple risk scores for a patient as well as benchmarks. It's unclear how readmission score is used in combination with other risk scores and may be hard to single out the actions as a result of readmission score. | Could be a concern It is designed to be deployed in EMR to generate patient-level readmission probability score to inform pharmacists' transition-of-care interventions between departments of medicine and pharmacy. It also requires a threshold score and is subject to concerns related to a single score as LACE and HOSPITAL. |
| ii) Might the use of the model perpetuate disparities even if the model’s predictions are accurate across groups? | No evidence/information | No evidence/information | No evidence/information | No evidence/information |
| iv) Might the model’s output lead to more uncertainty in decision making (e.g., if the model’s output is ambiguous)? | Not a concern  Model output has good interpretability and is straightforward. | Not a concern  Model output has good interpretability and is straightforward. | Not a concern  Model output has good interpretability and is straightforward. | Not a concern  Model output has good interpretability and is straightforward. |
